# Navigating socio-ecological and institutional barriers to antiretroviral therapy adherence: qualitative insights among young men and women from Nairobi’s informal settlements

**DOI:** 10.3389/fpubh.2025.1650966

**Published:** 2025-09-25

**Authors:** O. Muhenje, C. O. Olungah, D. O. Omia, R. O. Ondondo, P. Waswa, A. Lusambili

**Affiliations:** ^1^Institute of Anthropology, Gender and African Studies, University of Nairobi, Nairobi, Kenya; ^2^School of Public Health, Biomedical Sciences and Technology, Masinde Muliro University of Science and Technology, Kakamega, Kenya; ^3^Strathmore University Business School, Nairobi, Kenya; ^4^Environmental Health and Governance Centre, Africa International University, Nairobi, Kenya; ^5^NextGen For Earth, Nairobi, Kenya

**Keywords:** antiretroviral therapy, young people, adherence, retention, HIV, Kenya

## Abstract

**Background:**

The Human Immunodeficiency Virus (HIV) and acquired immunodeficiency syndrome (AIDS) pandemic remains a major global health issue, with 40.8 million people affected at the end of 2024. In Sub-Saharan Africa, Antiretroviral Therapy (ART) coverage reached 74%, though adherence remained challenging, particularly among youth due to poverty, stigma, and weak health systems.

**Aim:**

This paper explored socio-ecological and institutional barriers to ART adherence among young men and women aged 18–24 living in Kibra, Nairobi’s largest informal settlement.

**Methods:**

The study utilized phenomenological research design to explore lived experiences within social contexts to uncover hidden structural barriers using qualitative methods. In-depth interviews (*n* = 25), key informant interviews (*n* = 10), participant diaries (*n* = 25), structured clinic and home observations (*n* = 25), and case narratives (*n* = 10). Participants were purposively selected. Data were analyzed thematically using deductive and inductive coding in NVIVO 14.

**Results and discussion:**

Barriers emerged at individual, socioeconomic, and health system levels. These included limited ART knowledge, pill burden, comorbidities, food insecurity, stigma, violence, and negative healthcare provider attitudes. Addressing these requires multi-level interventions that go beyond medical treatment to tackle structural and social determinants of health.

## 1 Introduction

The Human Immunodeficiency and Acquired Immunodeficiency Virus (HIV and AIDS) pandemic remains a significant global health challenge. UNAIDS (1) statistics show that, despite global progress in HIV prevention and treatment, the epidemic remains marked by significant geographic and demographic inequalities. Africa continues to be the centre of the epidemic, with young people carrying a disproportionate share of new infections. In 2024, estimated 40.8 million people lived with HIV globally, and approximately 1.3 million new HIV infections occurred. Of these, the African region accounted for about 26.3 million cases—more than half of the global total. In 2023, an estimated 1.9 million young women (aged 15–24 years) and 1.2 million adolescent boys and young men were living with HIV worldwide. Strikingly, 77% of all new HIV infections among adolescent girls and young women occurred in sub-Saharan Africa (1). Non-adherence to HIV treatment results in an increased risk of drug resistance and treatment failure, which can lead to the deterioration of the immune system (2, 3).

Factors such as inadequate infrastructure, financial constraints, stigma, and lack of social support systems have been identified as significant barriers to accessing and adhering to ART (4). At a personal level, People Living with HIV (PLWHIV) can internalize negative attitudes from society resulting in feelings of self-shame, and worthlessness (5). Stereotypes and misconceptions regarding HIV at the community level exacerbate the burden of the HIV epidemic by fueling stigma, discrimination, and misinformation, which in turn hinder prevention efforts, delay diagnosis, and discourage treatment adherence (6). Generally, the attitudes and behaviors of discrimination and stigmatization from family members, community, and healthcare providers are driven by social perceptions, moral judgment, and or religious thoughts in the society where PLWHIV live (7). Socio-ecological barriers including perceived negative attitudes of healthcare providers and poor staff competencies also influence young people’s intention to use healthcare services (8).

In Lower Middle-Income Countries (LMICs), socio-ecological models (SEM) have been used in diverse settings to understand how an individual’s health seeking practices are influenced by social, cultural, environmental, economic, political factors at intra-personal, interpersonal and institutional levels of society. The SEM can be considered as a framework for conceptualizing, understating multi-level domains to participants experiences and perceptions of HIV/AIDS (9) as well as describing intersections between structural processes and interventions aimed at promoting societal norms. Individual, interpersonal, organizational and community related factors interact to shape the experience and influence engagement of young people with multilevel interventions (10).

Overall, Africa bears the greatest HIV burden globally with young people between 15–24 years being the most affected accounting for 2.2 million HIV cases in SSA as of 2021 (11). In Kenya, there has been a reduction in the number of new HIV cases by 68.4% from 101,448 in 2013 to 32,027 in 2021 thus lowering the prevalence from 6.04 to 4.25%. However, the significant burden of HIV in Kenya still exists among young people aged 15–24 years accounting for around 51% of new infections, an increase from 29% in 2013 in Kenya occurring within this age group, the burden being more pronounced among young women than young men (12). Informal settlements in Nairobi are among the most affected by HIV with a prevalence of 12%. Although high-risk sex remains a significant factor of the increase of HIV infections in informal settlements, limited access to service, pervasive poverty, and societal norms predispose young women to HIV infection risks more compared to their male counterparts (13). Young people are lagging behind in adherence and suppression of HIV/AIDS, especially in urban slums. There is also a significant research gap in understanding the socio-ecological and institutional barriers to ART adherence among young adults. Thus, this study sought to use the socio-ecological model as a framework to explore the barriers to ART adherence among young people in urban informal settlements in Kibra, Kenya.

## 2 Materials and methods

This study adhered to the Consolidated Criteria for Reporting Qualitative Research (COREQ) 32-item checklist (attached in Appendix 1) to ensure methodological rigor and transparency. The COREQ framework guided the reporting of key aspects including research team reflexivity, study design, participant selection, data collection, analysis, and presentation of findings. By following COREQ, we aimed to enhance credibility, reproducibility, and completeness of the qualitative methods employed, consistent with best practice standards for qualitative health research.

### 2.1 Study design

This study employed a phenomenological design within an exploratory qualitative approach to capture and interpret the lived experiences of young adults (18–24 years) living with HIV in Kibera. Phenomenology was appropriate because it focused on understanding participants’ subjective realities, meanings, and perceptions of ART adherence within their socio-ecological context. Through in-depth interviews, case narratives, constructionist diaries, and structured observations, the study sought to uncover how individual, interpersonal, institutional, and structural factors intersect to shape adherence behaviors from the participants’ own perspectives.

#### 2.1.1 In-depth interviews (IDIs)

In-depth interviews were conducted to explore thoughts, feelings, and experiences in adhering to ART. A sample size of 10 to 30 participants was suggested, with saturation likely after 15–20 interviews (14). This study reached saturation after 25 interviews, allowing for nuanced insights and improved data richness.

#### 2.1.2 Key informant interviews (KIIs)

Interviews were also conducted with clinical and non-clinical healthcare providers knowledgeable about ART adherence barriers among young people living with HIV/AIDS in Kibera. A sample size of 10 to 20 key informants was recommended (15). A sample of 10 participants was used to balance depth and diversity of insights while maintaining feasibility.

#### 2.1.3 Constructionist diary entries

Participants were asked to keep diaries on ART adherence barriers with a guide of recommended sample size of 5 to 15, with saturation likely to reach at around 10 diaries (16). A sample of 25 participants was used to collect longitudinal data and capture experiences over time (15).

#### 2.1.4 Case narratives

Participants gave in-depth, story-based accounts from their own perspectives. Saturation is usually attained with 5 to 10 participants (14, 17). A sample of 10 case narratives was used to collect detailed narrative data in this study.

#### 2.1.5 Structured home observation visits

The researcher visited participants’ homes to observe how their living conditions affected ART adherence. A sample of 20to 30 is recommended (18). A sample of 25 participants was used to gather insights into their living conditions (18).

#### 2.1.6 Structured clinic observation visits

Structured clinic observations are organized set ups where services are systematically offered by the healthcare providers (19). This method involved collecting data by observing same participants who took part in IDIs at the health facility. Although the recommended sample size for clinic visits is 20 to 50 participants, saturation is arrived at around 20 to 30 participants (20). As such, a sample of 25 participants was used to ensure efficient comparisons across participants (19).

### 2.2 Study setting

The study took place in Kibra Sub-County, Nairobi County, within the Kibera informal settlement, one of Africa’s largest slums. According to the Kenya Population and Housing Census, Kibra had a population of over 185,000 residents with over 61,500 households and a high population density (21). Consequently, some non-governmental organizations among other sources have estimated the population to be higher. For instance, the UN HABITAT report estimates the population between 500,000 to 700,000 with more than 2000 people per ha (22). In a different report, Kibra’s population was estimated to be between 700,000 to 1 million, highlighting that the numbers projected by the Kenyan government’s census were underestimated (23). These discrepancies still exist while most current studies estimate the population to be between 170,000 and 250,000 (24). There have been collaborative efforts to reduce the burden of HIV in Kibra. From 2016 to 2021, Amref Health Africa’s Kibera Reach 90 project aimed to reduce HIV transmission among PLWHIV in Kibra by enhancing accessibility to HIV/AIDS services at community level (25). During this period, Amref worked in collaboration with health facilities including Kibera Community Health Centre and provided different services among them enrolling HIV-diagnosed individuals on ART and offering psychosocial support. Community Health Volunteers and peer educators also distributed educational materials at community level to raise HIV/AIDS awareness. Shining Hope for Community (SHOFCO) is a community-based organization aimed at offering healthcare services in Kibra among them, providing preventive care and HIV care (26). Even with all these and other related interventions, the HIV prevalence and new HIV infections remain high with low adherence levels among individuals enrolled on ART.

### 2.3 Study population and sampling strategy

Participants were selected using a purposive sampling method focused on young people living with HIV (YPLHIV) in Kibra. The study began with selecting the Kibera Community Health Centre (KCHC) as the primary health facility due to its central role in providing HIV and tuberculosis care in Kibera. As the first facility in Kenya to introduce antiretroviral therapy (ART) in 2003, KCHC offers valuable insights into supporting young adults living with HIV in similar settings. Participants were identified using a line list from the facility’s Electronic Medical Records (EMR). The inclusion and exclusion criteria for young people living with HIV (YPLHIV) are summarized in Tables 1, 2.

**Table 1:** Inclusion and exclusion criteria YPLHIV.

Criteria	Inclusion	Exclusion
Age group	Young people aged 18–24 years	Below 18 and above 25
Duration of HIV treatment	Receiving HIV treatment in Kibra for at least 12 months before the study	Receiving HIV treatment for less than 12 months. Not residing in Kibra
Residence	Within Kibra	Outside Kibra
Treatment history	History of treatment defaulting but traced back on treatment, or consistently on treatment with no defaulting	

**Table 2:** Socio-demographics for the IDI participants (*n* = 25).

Participant characteristic	Range	Frequency	Average	Proportion (%)
Age	18–20	11	Mean age of 22 years	44
	21–24	14		56
Gender	Male	10		40
	Female	15		60
Residing with	Living alone	7		28
	Living with Parents	18		72
Marital status	Married	3		12
	Single	22		88
Level of education	Primary	4		16
	Secondary	16		64
	Tertiary	5		20
Years since diagnosis with HIV	0–10	14	9.2 years	56
	11–20	11		44
Years of ART treatment	0–5	15		60
	>5	10		40
Default history	Ever defaulted	11		44
	Never defaulted	14		56

The study focused on individuals aged 18 to 24 years to address the unique social, emotional, and economic challenges of this transitional phase and their impact on ART adherence. Participants with at least 12 months of HIV treatment were included to gain insights into long-term adherence factors, with a mix of those with consistent adherence and those with a history of treatment interruptions (IIT). This approach allowed for an exploration of experiences across stable and unstable treatment trajectories.

A sample of 5 clinical healthcare providers and 5 non-clinical healthcare providers with at least 6 months of experience in the Kibra community were also included to provide perspectives on the healthcare system’s role in addressing adherence challenges and socio-ecological barriers. From the electronic medical records (EMR), 148 active young people were purposively identified, 132 of whom had been on treatment for the past 12 months. Among them, 57 had a history of defaulting (19 males, 38 females), and 75 had no defaulting history (32 males, 43 females) Out of 132 eligible participants, a purposive sample of 25 was selected to ensure representation of key subgroups (e.g., defaulters and non-defaulters, males and females, varying treatment durations). Recruitment was guided by the principle of thematic saturation, a standard qualitative practice where sampling ceases once additional interviews no longer yield new information. This approach ensured methodological rigor by combining purposive sampling for diversity with saturation-driven recruitment, thereby strengthening the validity and trustworthiness of the findings. The same participants were followed up for more data collection through structured observations and personal constructionist diaries. From this sample size, 10 participants were selected for case narratives.

### 2.4 Consenting process

Before data collection commenced, the research team provided all participants with a detailed briefing on the study’s objectives to ensure their full understanding. This engagement aimed to promote openness and ensure participants clearly understood the study’s purpose and their involvement. Informed consent forms, available in Kiswahili and English, were either read aloud to participants who could not read in the In-Depth Interview (IDI) group or given as written documents to the key informants after which they were signed by the participants. These forms outlined the potential benefits of participation, ensuring adherence to ethical research standards. Written consent was obtained from all participants.

### 2.5 Ethical approval

Before data collection began, the study was approved by the Ethical Review Committee (ERC) of Kenyatta National Hospital and the University of Nairobi (reference number P/500/06/2021). Additional permission was granted by the National Commission for Science, Technology, and Innovation (NACOSTI) under license number NACOSTI/P/22/19231. The study further received ethical clearance from the Nairobi Metropolitan Services - Health Directorate’s Research Ethics Committee (REC) under clearance certificate number EOP/NMS/HS/199, and Kibra Sub- County, with approval from the facility in-charge.

### 2.6 Data collection

Data collection for this study spanned from September 2022 to September 2023 and employed multiple qualitative methods, including in-depth interviews (IDIs), key informant interviews (KIIs), participant diaries, and structured observations. Apart from KIIs, IDIs and Structured clinic observations that were conducted at the clinic, the rest were conducted in participants’ homes and their familiar environments for more privacy, confidentiality, and enhanced responses. Research tools were initially developed in English and translated into Kiswahili to accommodate the diverse population.

The research team participated in a three-day training facilitated by a female Principal Investigator (OM), a specialist in social behavioral sciences holding a master’s degree in population studies with research experience in HIV implementation programs. The sessions focused on building qualitative interviewing competencies through role-plays and pilot testing of the study tool that mirrored real-life interview situations. These activities enhanced the team’s ability to remain neutral, listen actively, and apply effective questioning techniques.

The interviews lasted 45 min to 1 hour, focusing on barriers to ART adherence, retention, stigma, and support systems. KIIs with healthcare providers explored similar themes, with interviews conducted in English. With participant consent, interviews were audio-recorded, and additional context were captured through notes. All interviews were held in a private setting to ensure confidentiality. Participants maintained diaries through the study’s request for over a month to document their daily experiences with ART adherence, including medication routines, side effects, and support systems. These diaries provided detailed insights into their challenges and coping strategies. Structured observations during home and clinic visits helped to capture unspoken barriers by assessing participants’ living conditions, medication routines, and support networks. In clinics, attention was given to patient-provider interactions, information dissemination about HIV, and adherence strategies, offering a comprehensive view of the challenges faced by young people living with HIV.

While data for interviews and observation were being collected, analysis was being conducted simultaneously to assess whether new insights or themes continued to emerge, and data collection was stopped after further analysis yielded no more new categories. This approach guided the sample size determination and ensured credibility and completeness of the results (27).

### 2.7 Data analysis

Audio recordings from the interviews were transcribed verbatim by the research team, with Kiswahili interviews translated into English for analysis. To ensure accuracy, transcripts were cross-checked against the original audio files. Unique identifiers were assigned to protect anonymity, and all data were securely stored on password-protected devices before being imported into NVIVO-14 for analysis.

The codebook was developed and revised throughout the process. Two independent coders, OM and PW conducted separate analyses, reaching consensus on the final themes to ensure rigor and reliability. Additionally, Braun and Clarke’s six-steps of thematic analysis were used. Audios were first transcribed verbatim, research assistants then immersed themselves in the data as they read and re-read through the transcripts to understand in depth and breadth of the content therein. Initial codes were then generated by the PW to label and organize the data in meaningful codes with full attention accorded to every data item. Code development was done deductively guided by the WHO’s five dimensions of adherence (28) and inductively to allow more sub-themes to emerge organically from the data under each theme. The research analyst and the principal investigator sort the codes into meaningful themes by synthesizing the data, identifying the relationship between codes and defining their properties. Visual aids like thematic maps were used to show how various themes were associated with ART adherence dimensions. Critical thinking and collaboration were significant as the coders debated the importance of different themes and sub-themes in line with the socio-cultural aspects on the in urban informal settlements.

Theme reviewing entailed identifying coherent patterns of the coded data and review of entire datasets. This led to collapsing of overlapping themes and re-working, renaming and refining others. This step was necessary to allow for validity and reliability of the selected themes. Themes were defined to ensure that the importance of participants’ experiences in the ART adherence context was well-captured. This phase involved synthesizing the insights attained from all datasets and presenting them in a manner that would be understood academically in across the community audience. This helped with clear presentation of the findings. Ultimately, a comprehensive report was produced. Quotes from participants were integrated under respective themes and sub-themes to offer a contextual analysis that connected the findings with the broader picture of socio-cultural challenges affecting ART adherence among YPLHIV in Nairobi’s Kibera informal settlement.

## 3 Results

### 3.1 Characteristics of IDI participants

Participants who took part in the IDIs provided their socio-demographic characteristics and they are presented in Table 2.

As shown in Table 2, a total of 25 participants were included in this study. For the years 21 to 24, comprised 14 participants while 11participants were between 18 to 20 years of age. The mean age of the participants was 22 years. Females were many by 15 and males were 10. 18 participants showed that they lived with their parents compared to 7 who lived alone. Additionally, there were 22 single participants than there were married participants who were 3. On the secondary education level only 16 participants confirmed this while 5 and 4 participants had tertiary and primary education levels, respectively. Between 0 and 5 years only 14 participants had lived with HIV while 11 had lived with HIV for more than 5 years since diagnosis. Finally, 14 participants had never defaulted while 11 of them had defaulted on ART at some point at the time of this study.

### 3.2 Characteristics of KII participants

The KII participants provided their demographic characteristics as presented in Table 3.

**Table 3:** Socio-demographic table for the KIIs (*n* = 10).

Participant characteristics	Range	Frequency	Proportion (%)
Clinical HCPs	Clinicians	2	20
	Counselor	2	20
	Nurse	1	10
Non-clinical HCPs	MIPA – Adult	3	30
	MIPA – OTZ	2	20
Gender	Male	5	50
	Female	5	50
Age	20–25	2	20
	26–30	2	20
	31–35	1	10
	35–40	2	20
	Above 40	3	30
Marital status	Single	1	10
	Married	6	60
	Separated	2	20
	Widowed	1	10
Years of service	0–5	1	10
	6–10	3	30
	11–15	4	40
	16–20	2	20
Area of residence	Within Kibra	5	50
	Outside Kibra	5	50

As presented in Table 3, the 5 clinical HCPs included 2 clinicians, 2 counselors, and 1 nurse while the non-clinical HCPs 5 included 3 MIPA-Adult and 2 MIPA-OTZ. There was equal gender representation of 5 males and 5 females. Above 40 years of age there were 3 participants while the age groups 20–25, 26–30, and 36–40 years had 2 participants each while 1 of participants was between 31 and 35 years of age. On years of service, 4 of the participants had 11–15 years of experience, 3 had 6–10 years, 2 has 16–20 years, and 1 had 0–5 years of experience. Finally, 5 of the participants lived within while 5 lived outside Kibra.

### 3.3 Themes and sub-themes for barriers to ART adherence

This study identified several factors to ART adherence. The classification of these factors was guided by the WHO dimensions of adherence. The main themes included: (1) patient-related factors; (2) Condition related factors; (3) Health system factors; (4) Socio-economic factors; and (5) Therapy-related factors as presented in Table 4.

**Table 4:** Barriers to adherence of young people living with HIV on ART.

No.	Theme	Sub-theme	Codes
1.	Patient related factors	Negative Patient Attitude	Negative attitude towards medication
			Forgetfulness, Oversleeping, Lack of Acceptance, strict school schedules, inflexible job schedules,
		Lack of ART knowledge	Myths and misconceptions, lack of knowledge and information
2.	Condition-related factors	Co-morbidities	MDR TB and HIV, Epilepsy and HIV
			Drug and Substance abuse and mental health
3.	Health system factors	Health facility factors	Proximity, Commodity stock-outs, Waiting times, Lack of privacy
		Healthcare workers attitude	Negative HCPs attitude, old HCPs, insufficient HCPs
		Transition from childhood to adults	Limited support
4.	Socio-economic factors	Poverty and Food Insecurity	Lack of food, lack of fare,
		Stigma and discrimination	Discrimination, Lack of privacy, fear of disclosure, negative effects of disclosure,
		Support structures	Lack of family support, storage structures
		Violence	Gender-based violence, intimate partner violence
5.	Therapy related factors	Pill burden	Drug size, Packaging/container, Frequency of taking pills, Side effects
		Drug fatigue	Feeling weak, dizzy, sleep

#### 3.3.1 Patient-related factors

Participants indicated that they had a negative attitude towards ART drugs. Also, participants highlighted that they did not have enough knowledge about ART which may have influenced their negative attitude towards the drugs.

##### 3.3.1.1 Negative patient attitude

Negative attitude as a barrier to ART adherence was linked to the “fear” that the client would take them for the rest their lives as one male respondent shared:

Quote 1: Negative patient attitude

*“Yes, some fear taking drugs/medicine. So, someone asks herself, “should I use these drugs for the rest of my life?” The person refuses getting linked to ART and says, “let what happens, happen.” (IDI_YFLWHIV_31_220808).*

Our findings show that daily life pressures can disrupt consistent ART. Several respondents observed how being occupied with work or other activities caused them to lose track of time and forget to take their medication. For some, once the scheduled time had passed, they opted to skip the dose entirely and resume the next day.

Quote 2: Patient factors

*“You may have a lot of work and time flies back and you forget. So, since the time is past, you decide to take the drugs the next day.” (IDI_YFLWHIV_14_220910).*

Quote 3: Patient factors (Forgetfulness)

*“Maybe, they can say that they were delayed at work, or they come tired from work, and they forget.” (IDI_YFLWHIV).*

##### 3.3.1.2 Lack of ART knowledge

Both patients and providers reported limited access to information about HIV as well as ART in the community. Belief was noted in the community about ART being associated with infertility among the users, as one young male respondent mentioned:

Quote 4: Infertility myths

*"Loss of libido is a misconception and that one I have experience about it. Most of men who are HIV positive will come with loss of libido before starting ART more so when someone is in stage 4, will come with loss of libido because of immune suppression but when we put them on ART, the libido comes back so it is a misconception but not due to drugs but I think due to other conditions. (KII_HCPC_F_23_080922).*

Quote 5: Myths (witchcraft)

*“Myth is that if you have HIV, you have been bewitched. Another myth is that if you have HIV, you are an outcast.” (IDI_YMLWHIV_19_220908).*

These *myths and misconceptions* were reported by both the young people and healthcare providers to have led to delays more often in initiating treatment, especially around ARVs and their impact. These have been reported below:

Quote 6: Myths (medication affects fertility)

*"Yes, there are those who say that once you start taking the medication, you won’t be able to get a child." (IDI_YFLWHIV_31_220808).*

Some key informants shared below some misinformation harbored by young people hindering their adherence.

Quote 7: Misinformation on ART

*"What makes them delay is the stories that they have heard about ARVs, like if you take ARVs you will be so fat... which is not true." (IDI_YMLWHIV_19_220908).*

Quote 8: Misinformation on ART makes them fat

*“What makes them delay is because of something in the stories they have heard about ARV is that you will be so fat, you will be so everybody will see you going to the clinic, you know, you will be subjected to erectile dysfunction (ED) which is not true…they are really so much concerned about it and like the group I have, they keep on asking me daily if that is the truth because right now, we have been changed from TLE to TLD and I hear that TLD is very bad, now you need to tell this client that DTG is one of the very best ARVs Kenyans I've ever had. So, you tell them that it is very quick in viral load suppression, such like stuff and if they tell you that they are worried about their weight, you take to the nutritionist for nutritional counseling like to eat in proportions and the rest.” (KII_HCPc_F_22_070922).*

##### 3.3.1.3 School and clinic schedule-related issues

Additionally, another barrier identified in this study was the school and clinic system schedules that conflicted with the participant’s time to adhere to their clinic appointments. Four study participants reported a lack of confidentiality and strict learning schedules often not allowing for timely clinical visits and fear of disclosure because of the stigma and discrimination that still exists in schools, which presented a huge barrier for adherence.

Quote: 9: School environment

*“Sometimes other schools do not understand and again, some parents fear, and they have not discussed with administration about the status of these children, even the child is denied permission, sometimes when the teachers are not aware….” (IDI_YFLWHIV_21_220909).*

Similarly, other participants shared.

Quote 10: Boarding schools as a barrier

*“Forgetting is an issue, like for school, for those who are in school, let’s say they are in boarding school and that time for taking his pills has arrived and he/she is late to school, and forgot to take his pills, he can’t go back, he misses his dose, and for those who have completed school let’s say busy schedules at work make them miss their doses.” (IDI_YFLWHIV_14_220910).*

Quote 11: School attendance vs missed clinic appointments

*“I took them for 3 months and again defaulted and by that time I had joined Zetech college and this was because the same time I was having my clinic appointments is the same time I was having either classes or exams and I was torn in between what to do, so I settle to do exams which runs for the whole week and that is how I postponed my clinic appointments and before you know it, you have defaulted a whole month, by the time you get to the hospital, everyone is so mad with you because you have again defaulted for both TB and HIV medication. Your file is marked in red because of your poor attendance and they told me that they have given me the last warning so if I repeat that they will not attend to me again, and that made me realize that I just need to be serious with my medication, so I fully adhered to TB medication and now I am on ART fully adhering.” (IDI_YFLWHIV_16_220916).*

#### 3.3.2 Condition related factors

Co-morbidities were mentioned by participants as barriers to ART adherence. They made these participants skip or stop medication.

##### 3.3.2.1 Co-morbidities

There were various co-morbidities pointed out by the participants and guardians including drug abuse, tuberculosis, mental health, and non-communicable disease. For instance, there was a case narrative of an epileptic male participant whose biological mother noted that on some occasions, he missed taking his drugs when he had seizures. His condition had not just affected him as an HIV client on ART in terms of adherence but also his mother who had to switch between careers. According to the mother, the costly epileptic drugs made him go without drugs leading to seizures which sometimes hindered him from taking ART drugs at the right time.

Quote 12: Physical and financial barriers

*“A 19-year-old male participant living with HIV and epilepsy, his biological mother described his routing as “a constant juggling act.” His mother left her formal employment to support him, a condition which interfered with daily lives. Limited finances meant that epilepsy medication was prioritized, resulting to missed ART doses. “Sometimes, my son skips his HIV pills because I must choose which one I can afford that week. At times, he gets seizure attacks and takes time to come around thus missing his HIV pills,” his mother shared. Cognitive fatigue and stigma have further isolated him from peers. This case highlights how overlapping health challenges, economic strain, and social stigma shape adherence.” (CN_4)*

Drug and substance use was another factor highlighted. According to one key informant, substance abuse might have caused the clients not to take their drugs as required. Some participants mentioned that tuberculosis hindered their HIV treatment as they were asked to stop ART and treat TB first before embarking on HIV treatment.

Quote 13: Substance abuse

*“May be the drugs he uses are the ones which confuse him not to take their medication. Also use or drinking alcohol. Alcohol can also make you forget. The injecting drugs which I have mentioned like cocaine, they are the ones which make them not to adhere to their medication, they don’t swallow their drugs on time. Someone becomes high.” (IDI_YMLWHIV_19_220908).*

Quote 14: Comorbidities

*“So, at that point I was diagnosed with both TB and HIV and they told me because you had already started the medication, you will have to stop first so that we treat TB then after I will go back to HIV treatment. I started again asking myself who is going to becoming here for the next 6 months and the way it is far from home.” (IDI_YFLWHIV_13_220909).*

Quote 15: Substance abuse (2)

*“Maybe he is addicted to drugs, and he has found out his status, it will be hard because he will be smoking bhangi (weed) while going for medication.” (KII_HCPnc_F_38_060922).*

#### 3.3.3 Health system factors

##### 3.3.3.1 Health facility factors

The study participants frequently mentioned long distance, poor road network and lack of fare as some of the major barriers to accessing care. Given the urban informal settlement nature of the study area, accessibility was noted to be a problem especially during rainy season. This was because most of the patients in the informal settlement utilized motorbikes or went to facilities by foot as seen in the clinical observations. 5 participants mentioned that they lacked fare and the fact that some had not disclosed their status to their parents/guardians made it even harder. This was shared by a female respondent below:

Quote 16: Distance to clinics

*“…distance also becomes a hinderance because some youths may not be able to afford fare and due to the fear of disclosing this to the parents, knowing they won’t get support to attend appointments also becomes a major reason.” (KII_HCPC_F_11_220907).*

In addition to the distance to the facility, the young people reported processes at the facilities being long thus perceived to waste their time. This was noted from clinical observations and reiterated in the IDIs as shared by a young female living with HIV:

Quote 17: Clinic time as a barrier

*“…issues of always going (to a health facility) is a waste of time. Like you see the way it happens with TB patients, you just come, weigh, and take your drugs and go back home. There are no other things like you having to take your pressure and queue for another hour to take your weight (before being given the drugs).” (IDI_YMLWHIV_15_220915)*

Quote 18: Clinic waiting time

*“Generally waiting time for Outpatient (OPD) and Comprehensive care clinic (CCC) clients normally about 20 mins before they are called by the doctor. They take about one and a half hours during the clinic visit.” (KII_HCPnc_37_070922).*

Other barriers reported by the young people were lack of privacy at the facilities and breaching patient confidentiality. The young people reported to be uncomfortable being attended to by older health care providers (HCPs), leading to poor retention of young people on ART as reported by a clinical healthcare provider:

Quote 19: Self stigma

*“maybe there's somebody at the bench that they are afraid of, so such people might be a big hinderance for them to pick the drugs, for instance if you ask them why they don’t want to go to the pharmacy, they will tell you there is a woman who is a friend to my mum and I don’t want her to see me, so just do the courtesy of picking for me the drugs.” (IDI_YMLWHIV_19_220908).*

Quote 21: Patient fear

*“What I would like to change is that we are mixed with other patients who are not on ART. Those of us who are on ART have a specific card that we place on the table when we arrive. This makes others wonder why we have the card and they don't, which creates suspicion.” (Diary Entry 19).*

Quote 21: Fear of unintended disclosure at the clinic

*“The waiting area is shared by everyone both general OPD patients and those attending the C.C.C. There is no dedicated space for adolescents. They (young people) feel uncomfortable with this arrangement given they wait for a long time to be called in by the doctor.” (Structured clinical observation 5).*

##### 3.3.3.2 Health care workers attitudes

Negative patient-provider attitude was mentioned as being a barrier towards ART adherence. A clinical healthcare provider and a young person recounted:

Quote 22: Healthcare worker attitude

*"If they (young people) get a healthcare worker with an attitude, it will influence them on how they take their ART, they will not keep their appointments, As we've said, this is a lifelong treatment, so it's essential to be positive and empathetic. The first interaction is critical it can determine whether a person decides to begin treatment. It's also important that you are not judgmental during that initial encounter." (KII_HCPnc_37_070922).*

Quote 23: Healthcare workers’ negative attitude

*“Some insult you like you find that maybe someone came with anger from their house when they find you there, they talk to you harshly asking you are they the ones who send you and such things really demoralize you because you even don’t know where you got it from.” (IDI_YMLWHIV_17_220908).*

Similar sentiments from other clinical health providers were noted:

Quote 24: fear of health provider judging them

*“So, maybe some youths might think that if you are dealing with elderly providers, they are more entitled to judging you than when you are dealing with somebody who is closer to your age.” (KII_HCPc_34_060922).*

Contrary to the above, a female healthcare worker respondent added that:

Quote 25: Youth to lead support group

*“Counselling support groups for the youths living with HIV, should always be done by fellow youths living with HIV. That would help so that every time there’s a group meeting, they will attend and continue adhering to ART treatment.” (KII_HCPc_22_070922).*

Similar sentiments from other clinical health providers were noted:

Quote 26: Healthcare workers influence adherence

*"If they (young people) get a healthcare worker with an attitude, it will influence them on how they take their ART, they will not keep their appointments, which will make them have high viral load, which will influence their health negatively." (KII_HCPnc_37_070922).*

Stock-outs and insufficient health care providers were also highlighted. This was coupled with the general youth health-seeking behavior, which was reported to be “last minute.” A young female reported:

Quote 27: Number of healthcare workers

*“The barriers are maybe if the doctors are not enough, maybe the one dealing with issues concerning HIV is not around, maybe there are not enough drugs.” (YMLWHIV_10_220817).*

#### 3.3.4 Socio-economic factors

The study participants also reported poverty and food insecurity, lack of privacy at homes, stigma and discrimination, fear of disclosure, lack of support structures, SGBV and IPV as some of the barriers to adherence to HIV treatment among the young people living with HIV.

##### 3.3.4.1 Poverty and food insecurity

Patient decisions to adhere to HIV treatment were also impeded by poverty and food insecurity. Young people reported that they could not take drugs at the required time due to lack of food as taking drugs on an empty stomach often led to them feeling dizzy and nauseated as noted by a young female living with HIV.

Quote 28: Home situations affect adherence

*“You will find that there’s someone who may lack food and cannot take his/her medication on an empty stomach because sometimes when you take the drugs, they make you feel dizzy. So, she/he says, “when I take drugs, they will make me feel bad, so let me just stay until the time I get something to put in the stomach, that is the time I will take.” (IDI_YFLWHIV_20_220808).*

Quote 29: medication side effects

*"If I take my medicine without eating, this medicine is very strong in which case the medicines make me feel dizzy and I get very weak." (Diary Entry_ YMLHIV).*

In a case narrative with a 19-year-old female participant living with HIV, with her HIV positive husband and HIV negative son, she described frequent struggles with food insecurity. Irregular income from her husband’s unstable jobs often left them without adequate meals, making it difficult to take ART consistently. Their experience illustrated how poverty and nutritional challenges intersected with health behaviors, underscoring micro-level barriers to adherence and the need for integrated support that addresses both food and treatment access.

Quote 30: Lack of food

*“It’s hard to take medicine on an empty stomach.” (CN_1).*

Poverty was also associated with young people failing to continue with school. This in turn was also reported to have a negative effect on adherence to ART. A respondent reported that there was no need of taking drugs if they were not going to school.

Quote 31: Lack of food

*“… social economic status, this may make her start thinking negatively towards even the treatment, if I cannot go to school, there is no need of taking my drugs.” (KII_HCP_C__F_11_220907).*

Apart from poverty as a crucial factor to adherence to ART, most participants also expressed challenges related to transitioning from youth to adulthood being a factor to treatment adherence. This was because this transition came with additional responsibilities, especially financial needs, which is always stressful. This was key because they were now considered as adults thus perceived to be able to take care of themselves. Failure to a timely report to healthcare facilities for clinical visits was reported to be caused by a lack of fare or strict job schedules. This was reported by a clinical health provider:

Quote 32: Socio economic status

*“… possible that compared to infants and young children, adolescents and youth are seen as more independent and are therefore, less likely to receive the support needed as they transition towards adulthood”. (KII_HCPc_33_060922).*

##### 3.3.4.2 Lack of privacy at homes

Concerns about unintended disclosure of young people’s HIV status was a barrier to exposing their HIV status as they did not want people to know it. This was indicated below in the different quotes by the different respondents:

Quote 33: Lack of support

*“You know that one has a challenge because we are groups and we are discussing in groups. You know each person wants to keep it secret, you know I may be using the ART medication, and I don’t know the other person when he takes the medication.” (IDI_YMLWHIV_19_220908).*

Quote 34: Lack of phone affects getting reminders

*“The residence of the most of young people are made of muddy walls with mabati (iron sheets) roofing. For most of the young people taking ARVs, when they are reminded by their guardians or parents to remember to take their ARV medicines, the neighbors do hear and unintended disclosure of their HIV status occurs, fueling a lot of stigma.” (Home visit_6).*

Quote 35: Housing situation leads to unintended disclosures

*“So it depends on if they are sharing a phone with a family member whom they have not disclosed to, they might not want that but if it is their own personal phone, then they accept.” (KII_HCPc_F_22_070922).*

##### 3.3.4.3 Stigma and discrimination

Stigma and discrimination were presented at different levels with most sentiments revolving around personal beliefs and internalized stigma, discrimination social cycles such as families, at the community level, and to a small extent at the organizational level particularly schools and healthcare centers. Nine participants mentioned that after being diagnosed with HIV, they felt undeserving thus developing internalized guilt and shame. They felt stigmatized and discriminated not because they had been victims before but because they had seen other people in society going through the same “shame.” This limited disclosure which in some cases was done unintentional at both intrapersonal and community levels, making it hard for them to relate with family and friends. Stigma due to unintended disclosure was reported to affect the young people of both genders. Female participants also noted that young women who were diagnosed with HIV were in most cases perceived to be promiscuous and the criticism extended from families through the community to healthcare providers.

These sentiments were shared by several respondents as stated below:

Quote 36: Community stigma

*“…at the community level, there are many things like being discriminated against by others … and this may lead to him/her losing friends … it makes it difficult for them to have a close relationship between him and his family.” (IDI_YMLWHIV_20_220808).*

Quote 37: Fear of carrying ARVs home

*“Someone may fear using (ARVs) because once they are given, they will carry them to the house, and it is something that cannot be hidden … They discriminate you, especially from other people when it comes to sharing of utensils.” (IDI_YFLWHIV_31_220808).*

Quote 38: Discrimination in schools

*"There was a young person who told me that when they were in a boarding school and one of his friends was discovered to be HIV positive… he started being discriminated … so he was also afraid of starting on ART treatment." (KII_HCPc_F_22_070922).*

In a case narrative with a 20-year-old male living with his mother who was a commercial sex worker, his HIV was unintentionally disclosed to his friends and neighbors by his biological mother and a healthcare who reminded him to take ARVs and questioned why he missed previous clinics, respectively. Living with a single mother who was a sex worker, he faced intense stigma and isolation, disrupting his ART adherence. With support from the study team and peer groups, he gradually re-engaged with treatment, illustrating the critical role of community networks and psychological support in overcoming stigma and improving health outcomes.

Quote 39: Stigma at the community

*“Everyone in the neighborhood knew before I even told them.” (CN_2).*

Another young person shared their experience on stigma and discriminations as well:

Quote 40: Discrimination of other students

*“When you find that it is in classroom or you are participating in some activities, you are removed because they know, you are HIV positive.” (IDI_YFLWHIV_31_220808).*

Respondents also reported that discrimination always led to fear of disclosure of one’s status to evade rejection, blame from sexual partners and isolation by peers thus adversely affecting their adherence and retention. These sentiments were shared by one peer educator:

Quote 41: Negativity from friends

*"Friends are the worst virus than the HIV virus itself because they will tell you all the negative stuff about HIV and ARVs." (KII_HCPnc_37_070922).*

Quote 42: Unintended disclosure

*"Today, a friend of mine came across my medication and started asking what kind of medicine it is and what it’s for. I haven’t responded yet because I don’t know what to say. I’m worried she might look it up online and figure it out, so now I’m left wondering should I tell her myself, or wait for her to find out on her own?" (Diary Entry 24).*

In addition to stigma and discrimination, some of the young people and HCPs also reported the existence of gender-based violence and intimate partner violence in the community. As a clinical healthcare provider and a young male living with HIV shared:

Quote 43: IPV poses a challenge

*"For barriers, there is intimate partner violence. You may encourage them (young people) to disclose, but for example, if they disclose their HIV status to their partner, they may start fighting over such things." (KII_HCPc_F_33_060922).*

Quote 44: Intimate partner interruption

*“I woke up this morning and before I took my medicines my husband interrupted me by starting to quarrel me, you know he does not want me to take my medicines. That made me delay taking my medicines.” (Diary Entry 20).*

Yet another young female living with HIV shared her experience on Intimate Partner Violence (IPV) in a case narrative following disclosure of HIV status as well.

Quote 45: Partner violence and stigma

*"He (boyfriend) used to beat me, when we disagree sometimes, he would take the medicine and pour them which is embarrassing.” (CN_YFLWHIV's).*

Discrimination and violence acted as a significant barrier to disclosure as reported by young people thus this was an overall barrier to adherence. Additionally, young people who were MSM highlighted that they were being stigmatized for being MSM and HIV positive at them same time as one young male reported:

Quote 47: Homophobic stigma by community

*"If I were to go and tell him right now, he might even kill me, he will ask me, 'Why I didn’t tell him earlier?'" (CN_YMLWHIV, MSM).*

*We were labeled and considered as demons-possessed persons no one who knew about our relationship could rent out house unto us till we're forced to shift.” (CN_YMLWHIV, MSM).*

*“At the facility some staffs knew about our affair, during one of my visits I would be escorted by my partner and rarely would HCW see us as a couple.” CN_YMLWHIV_MSM).*

In yet another case narrative on sexual violence and adherence, with a 19-year-old male living with perinatally acquired HIV, he shared that he endured repeated sexual and emotional abuse from his biological mother, who demanded sex in exchange for food. Trapped in a one-room home and unable to leave or form a good relationship with the mother, his ART adherence suffered, and his viral load increased. Intervention by health professionals enabled him to move in with peers and regain some stability. This case underscored how sexual violence, even within families, can severely undermine mental health and adherence, requiring urgent protective interventions. The young man reported that this sexual abuse had a significant negative impact on his adherence, missing his medication and interrupting treatment and retention and a clinical healthcare provider who served him also reported on the same:

Quote 48: Food for sex

*“You must have sex with me before you eat.” (CN_3).*

Quote 49: Family stigma and alcohol

*“The mother is always drunk and shouts at the boy, hey you, come and have sex with me, you cannot eat my food free in this house, food I have worked hard to get, and you don’t fuck me, there is nothing for free here.” (KII_HCPC_F_11_220907).*

#### 3.3.5 Therapy related factors

##### 3.3.5.1 Pill burden

Pill burden was frequently mentioned by respondents in both IDIs and KIIs as a barrier to adherence. These were captured by the sentiments from a young male living with HIV:

Quote 50: The ARV tablets size

*“The size is so big before it goes down the throat, and it’s not chewable you just need to swallow it and drink some water for it to go down, it takes some time.” (IDI_YMLWHIV_15_220915).*

Yet another young female respondent also reiterated,

Quote 51: Fear of carrying the tablets

*“We are given drugs so that we can go back to school. I took all those tablets at once, but I felt so much nausea before swallowing them and I did vomit all of them and they also turned so bitter in my mouth and I just spit all of them, then again, I tried mixing all of them, then the time I wanted to drink, I can’t tell what happened but the glass fell off.” (IDI_YFLWHIV_20_220808).*

Informants noted that the current practice of prescribing one tablet a day had lessened the burden compared to the previous practice where multiple tablets were prescribed. As recounted by one female respondent:

Quote 52: One tablet a day is better than 3 times a day

*“Some taking the drug once a day is easier to accept unlike telling them to take thrice a day which becomes difficult.” (YMLWHIV_10_220817).*

Consistently, a health care provider recounted.

Quote 53: Pill burden

*“If it is a lot of tablets, they are more likely to refuse but if it is just one tablet it becomes simpler for them to take in a day.” (KII_HCPc_22_070922).*

##### 3.3.5.2 Drug fatigue

Drug fatigue and skepticism was reported by young people living with HIV as a barrier to adherence this is reported by a young female living with HIV:

Quote 54: Medication fatigue

*“Maybe someone may experience fatigue in taking the drugs. ARVs are drugs which you take every day, and someone may just feel fatigued in taking them. The drugs have side effects, initially when you start, you may feel nauseated, vomiting and, dizzy, which makes someone to get bored. Therefore, the person may just decide not to continue with the drugs.” (KII_HCPc_22_070922).*

Quote 55: Tired of medication

*You see, sometimes one gets fatigued taking medication every day. You just say, let me stop for some time, I will resume later.” (IDI_YMLWHIV_20_220808).*

Quote 56: Feeling tired and isolated

*“The young person sometimes gets skeptical after being on ARVs for a long time and getting tired of taking the ARV medicines everyday (drug fatigue) and as well having feelings of shame and isolation by his friends as well as gossip in the community because of his HIV status (self-stigma). (KII_HCPc_34_060922).*

##### 3.3.5.3 Hallucinations and lipodystrophy

Another effect associated with HIV treatment was hallucinations and lipodystrophy. As highlighted by one HCP clinician, most young people especially females were really concerned about issues related to Lipodystrophy. Clinicians also mentioned experiences of young people that were causing depression.

Quote 57: Drugs side effects (Weight)

*It's not so much for young youth male, but it's youth female. They are really concerned about the weight because it's for sure that it has something that will have some add weight and that one is for a fact.” (KII_HCPc_33_060922).*

Quote 58: Drugs side effects

*“It had a lot of lipodystrophy issues. And efference it had issues with hallucinations. Young people are saying once they have taken the drug, they sometimes start hallucinating that they are burning in the house then he or she starts screaming then when the neighbors ask them why they were screaming does it mean that you are taking the ARVs drugs, so you become uncomfortable.” (KII_HCPc_33_060922).*

The above barriers identified by the young people and Key informants in this study are summarized in the Socio Ecological diagram in Appendix 2.

## 4 Discussion

This study identified key factors that influence and affect adherence and retention of young people living in informal settlements on HIV treatment. The socio-ecological model (SEM) was used in this study as a comprehensive framework for understanding ART adherence by recognizing the complex interplay between individual, interpersonal, community, institutional, and policy-level factors (29). While adherence is often framed as an individual responsibility, this model highlighted how broader social and structural determinants significantly impacted a person’s ability to remain on treatment. Applying SEM to WHO’s five dimensions of ART adherence revealed that barriers exist at multiple levels, from personal struggles with self-acceptance and internalized stigma to healthcare system inefficiencies and socio-economic constraints. By mapping these dimensions onto SEM, it became evident that interventions must go beyond individual behavior change and address structural and systemic challenges to improve ART adherence among young people.

### 4.1 Patient-related factors

Key barriers identified in this study were negative patient attitudes toward medication and a lack of knowledge about ART. Young people reported feeling overwhelmed by the lifelong nature of ART, leading to treatment fatigue and occasional non-adherence. This aligned with findings that young people struggle with accepting their HIV-positive status, which, in turn, affected ART adherence (30). In line with this, a study in Uganda found that self-acceptance challenges contributed to increased internalized stigma, which can deter consistent ART use (31). The finding that internalized stigma among young people interfered with ART adherence echoed previous research indicating that young people were more likely to experience internalized stigma than older adults (32). At the individual level of SEM, these barriers stemmed from personal beliefs, emotions, and knowledge gaps.

However, at the interpersonal level, societal stigma and peer dynamics played a role, as YPLWHIV absorbed negative societal attitudes and imposed them on themselves, leading to shame and fear that significantly impacted adherence. Other individual-level barriers included forgetfulness and oversleeping, compounded by the fact that many young people in this study were forced into financial independence at 18 years, working menial jobs that left them exhausted and more prone to missing doses (30). With SEM’s community level, studies in Uganda and Zambia have found that myths, misconceptions, and limited ART knowledge negatively impacted adherence (33). Additionally, school and job schedules had been identified as adherence barriers in Tanzania and Uganda which are consistent with prior findings (34, 35).

### 4.2 Condition-related factors

This study found that comorbidities such as tuberculosis (TB), mental health conditions, and non-communicable diseases affected ART adherence. Young people reported discontinuing ART when diagnosed with multi-drug-resistant tuberculosis (MDR-TB), as they were advised to prioritize TB treatment. This finding was consistent with research indicating that co-treatment for HIV and TB presented adherence challenges, as overlapping drug side effects may lead patients to discontinue one or both treatments (36). At the organizational level of SEM, the lack of integrated HIV-TB care in many healthcare facilities exacerbated these issues.

Similarly, epilepsy was identified as another condition affecting adherence, with the high cost of epilepsy medication being a significant burden, especially in informal settlements where poverty is widespread. The absence of epileptic drugs increased seizure frequency, which in turn disrupted ART routines. These findings were aligned with prior research showing that beyond drug to drug interactions, seizures particularly recurrent ones impaired memory and ART adherence (37, 38). Additionally, alcohol and substance abuse were found to be major adherence barriers, often initiated due to peer pressure, a finding echoed in previous studies (31, 34, 39). Consistent with studies in Rwanda, mental health conditions such as depression and substance use disorders had been strongly linked to ART non-adherence (40). At the policy level, integrating mental health and substance abuse services within HIV programs could improve adherence among affected youth.

### 4.3 Health system-related factors

Health system-related barriers included long distances to clinics, ART stockouts, long waiting times, shortages of healthcare workers, fixed clinical appointments, and privacy concerns. Many young people reported that they did not receive adequate counseling due to healthcare worker shortages, which negatively impacted their understanding of ART. Findings from South Africa, where low patient-provider ratios resulted in insufficient counseling and increased misinformation (41). Long waiting times at clinics also deterred many young people from accessing services, particularly those who were employed or in school, which was consistent with studies from sub-Saharan Africa (34, 41).

At the community level, long travel distances, long waiting hours, and favoritism on who is served first at the CCC clinic were reported in this study as barriers to adherence, echoing findings in a study conducted in Uganda have (42). Healthcare workers’ negative attitudes were also reported as a barrier, with some young people citing experiences of judgment and lack of youth-friendly services. These findings echoed research in Tanzania, where negative provider attitudes discouraged young people from seeking HIV treatment (35). At the organizational level, the patient-provider relationship played a crucial role in adherence, and when healthcare professionals failed to provide adequate support, young people may disengage from treatment. Similar studies highlight that training healthcare workers on youth-friendly services and increasing the number of professionals in high-burden areas could improve retention (34). Training healthcare workers on youth-friendly service provision and increasing the number of trained professionals in high-burden areas could improve ART retention.

### 4.4 Socio-economic factors

Poverty and food insecurity were found to be major socio-economic barriers to ART adherence. Many participants struggled with a lack of food and transport costs, making it difficult to attend clinic appointments or take medication consistently. Studies in Uganda found that food insecurity led to poor adherence, as individuals feared taking ART on an empty stomach due to side effects (43). Stigma and discrimination were also significant barriers, with young people fearing disclosure and avoiding medication in public. A study in Zimbabwe highlighted that these findings, demonstrating that stigma led to ART non-adherence among young people (44). At the interpersonal level, young people also reported experiences of breaches in confidentiality by healthcare providers, leading to missed clinic visits. Consistent with research in sub-Saharan Africa, fear of stigma and discrimination remains a major reason for ART discontinuation. Stigma was even magnified in the school setting with students refusing to share essentials with those reported to be HIV positive (45). Similar research in Sub-Sahara Africa revealed that among the reasons why young people may discontinue ART treatment was due to fear of disclosing their HIV status to avoid potential stigma and discrimination (46, 47).

Lack of support structures both at home and in schools was reported to be a major barrier. In schools, the barriers were mainly related to privacy and storage facilities, including the absence of structures to compensate for overlaps between clinic appointments and school activities. At the community level, the school environment played a crucial role in shaping young people’s adherence behaviors, yet many educational institutions failed to provide a supportive framework for YPLHIV. Additionally, violence—particularly gender-based and intimate partner violence (IPV) was identified as a socio-economic barrier to ART adherence. Young women, in particular, faced heightened risks of violence, making it difficult to consistently take their medication (48). This aligned with findings that survivors of sexual and gender-based violence (SGBV), whether from biological parents or intimate partners, often struggle with ART adherence due to emotional trauma and disrupted routines. Previous studies on SGBV and IPV have been strongly associated with reduced adherence among adolescents living with HIV (49). These findings underscored the need for school-based interventions, community support structures, and trauma-informed care to mitigate the impact of violence on ART adherence.

### 4.5 Therapy-related factors

Pill burden and drug fatigue emerged as significant therapy-related challenges. Many young people reported difficulties with pill size, packaging, frequency of intake, and side effects, such as dizziness, fatigue, and headaches, which discouraged adherence. These findings aligned with research in Malawi, where pill burden was a common barrier among adolescents transitioning to adult HIV care people (39). Research in Malawi showed that pill burden discouraged adherence, especially among adolescents transitioning from pediatric to adult care (50). Drug fatigue, where individuals become exhausted from taking lifelong medication, was a well-documented challenge in long-term HIV treatment (51). Furthermore, pill burden, drug fatigue, side effects, and skepticism reported by study participants was a barrier to ART adherence were consistent with other studies in sub-Saharan Africa with the youth just getting tired of taking the ART medication leading to them taking “drug breaks” from medication which was been associated with the development of resistance to drugs (31, 35). Drug resistance requires more complex regimens to suppress the virus which has been reported to be costly (52).

## 5 Conclusion

This study, conducted in Kibra, an informal settlement, provided crucial insights into the barriers affecting ART adherence among young people, expanding on previous research through the Socio-Ecological Model (SEM) and the WHO’s five dimensions of ART adherence. The individual-level findings aligned with the patient-related dimension, highlighting how conditions like epilepsy disrupt ART adherence not only due to drug–drug interactions but also because seizures impair memory and disrupt daily routines. At the interpersonal level, financial struggles were a major barrier, particularly for young people who rely on physically demanding menial jobs that leave them too exhausted to take their medication consistently. At the institutional level, health system barriers were prominent, with breaches of confidentiality by healthcare providers discouraging treatment retention. At the policy level, findings reveal that Kibra’s school environments lack essential support structures for young people on ART, such as safe medication storage and flexible schedules for clinic visits. Overall, this study demonstrated that ART adherence among young people in Kibra was shaped by a complex interplay of individual, social, economic, and structural factors, as captured in the Socio-Ecological Model and WHO’s five dimensions of adherence. Addressing these barriers required a multi-level approach, including strengthening healthcare systems, providing socio-economic support, integrating mental health care, and implementing school-based interventions. The findings emphasized that improving ART adherence in informal settlements requires interventions that go beyond medical treatment to address broader social and structural determinants of health.

The study strengths included the Method triangulation approach which was employed in this study and is essential in future research in understanding complex issues surrounding the adherence challenges and experiences of young people living with HIV in urban informal settlements as well as other settings. The limitations included use of purposive sampling from one main comprehensive health facility and the small sample size which limited the generalizability of the findings to other populations and geographical regions.

## Data Availability

The raw data supporting the conclusions of this article will be made available by the authors, without undue reservation.
