# Supplementary material for: Navigating socio-ecological and institutional barriers to antiretroviral therapy adherence: qualitative insights among young men and women from Nairobi’s informal settlements
Source: Front Public Health. 2025 Sep 25;13:1650966. doi: 10.3389/fpubh.2025.1650966 (PMC12507891; doi:10.3389/fpubh.2025.1650966)
Supplement: Supplementary file 1 [file Supplementary_file_1.docx]

# APPENDICES

## Appendix 1: COREQ Checklist (32 items) for the Manuscript: “Navigating Socio-Ecological and Institutional Barriers to ART Adherence”

This study adhered to the Consolidated Criteria for Reporting Qualitative Research (COREQ) 32-item checklist to ensure methodological rigor and transparency. The COREQ framework guided the reporting of key aspects including research team reflexivity, study design, participant selection, data collection, analysis, and presentation of findings. By following COREQ, we aimed to enhance credibility, reproducibility, and completeness of the qualitative methods employed, consistent with best practice standards for qualitative health research.

| **Domain** | **Item** | **Guide Questions** | **Response for this Manuscript** | **Page** |
| --- | --- | --- | --- | --- |
| **Domain 1: Research Team and Reflexivity** | | | | |
| Personal characteristics | 1. Interviewer/ facilitator | Who conducted the interviews/focus groups? | Principal Investigator (PI) conducted most interviews, assisted by trained research assistants. | p. 12 |
|  | 2. Credentials | What were the researcher’s credentials? | PI holds a Master’s in Population Studies; team trained in HIV research and anthropology. | p. 12 |
|  | 3. Occupation | What was their occupation at the time? | PI is a Behavioral Scientist and currently a PhD Student in Anthropology at the Institute of Anthropology, Gender and African Studies (IAGAS) at the University of Nairobi, Kenya. | p. 12 |
|  | 4. Gender | Was the researcher male or female? | PI: Female; team included both genders. | p. 12 |
|  | 5. Experience and training | What experience/training did the researcher have? | PI experienced in HIV research; team trained in qualitative interviewing, neutrality, and piloting. | p. 12 |
| Relationship with participants | 6. Relationship established | Was a relationship established prior? | Yes. Rapport built via clinic visits and study briefings. | p. 12 |
|  | 7. Participant knowledge of interviewer | What did participants know about the researcher? | Participants informed about study purpose and PI’s HIV focus. | p. 12 |
|  | 8. Interviewer characteristics | What characteristics were reported? | PI’s social-behavioral expertise, gender facilitated rapport. | p. 12 |
| **Domain 2: Study Design** | | | | |
| Theoretical framework | 9. Methodological orientation/theory | What theory underpins the study? | Socio-Ecological Model (SEM) and WHO’s five adherence dimensions guided analysis. | p. 5–6 |
| Participant selection | 10. Sampling | How were participants selected? | Purposive sampling from Kibera Community Health Centre EMR. | p. 10–11 |
|  | 11. Method of approach | How were participants approached? | In-person via clinic records, CHVs, and peer educators. | p. 10 |
|  | 12. Sample size | How many participants? | 25 IDIs, 10 KIIs, 25 diaries, 25 observations, 10 case narratives. | p. 6, 10–11 |
|  | 13. Non-participation | How many refused/dropped out? | None |  |
| Setting | 14. Setting of data collection | Where was data collected? | Kibera informal settlement: clinic CCC, homes, and community. | p. 9 |
|  | 15. Presence of non-participants | Anyone else present? | Family members occasionally present during home visits. | p. 12 |
|  | 16. Description of sample | Key characteristics? | IDIs: Age 18–24 (mean 22), 60% female; KIIs: clinicians, counselors, peer educators (50% male/female). | p. 18–19 |
| Data collection | 17. Interview guide | Questions provided/pilot tested? | Yes. Developed in English, translated to Kiswahili, piloted and revised. | p. 12 |
|  | 18. Repeat interviews | Were repeat interviews done? | No repeat IDIs; diaries/observations provided longitudinal data. | p. 12 |
|  | 19. Audio/visual recording | Was data recorded? | Yes, audio-recorded with consent. | p. 12 |
|  | 20. Field notes | Were field notes made? | Yes, during interviews, clinic, and home observations. | p. 12 |
|  | 21. Duration | Interview duration? | 45–60 minutes per IDI/KII; diaries over 1 month; observations multiple hours/days. | p. 12 |
|  | 22. Data saturation | Was saturation discussed? | Yes, saturation reached at 25 IDIs, confirmed during analysis. | p. 12 |
|  | 23. Transcripts returned | Were transcripts returned? | No transcript return; |  |
| **Domain 3: Analysis and Findings** | | | | |
| Data analysis | 24. Number of data coders | How many coders? | Two coders (OM & PW). | p. 13 |
|  | 25. Description of coding tree | Coding tree described? | Deductive (WHO) + inductive codes refined iteratively; thematic maps used. | p. 13 |
|  | 26. Derivation of themes | Themes pre-identified or emergent? | Combination of pre-identified (SEM) and emergent from data. | p. 13 |
|  | 27. Software | What software used? | NVIVO 14. | p. 13 |
|  | 28. Participant checking | Did participants check findings? | No formal member checking |  |
| Reporting | 29. Quotations presented | Were participant quotations used? | Yes, anonymized quotes (IDI, KII, diaries, case narratives). | p. 18–45 |
|  | 30. Data and findings consistent | Data consistent with findings? | Yes, triangulated across multiple qualitative methods. | p. 18–47 |
|  | 31. Clarity of major themes | Major themes clear? | Yes: 5 main themes (patient, condition, health system, socio-economic, therapy). | p. 20–46 |
|  | 32. Clarity of minor themes | Minor themes discussed? | Yes: GBV, stigma in schools, lipodystrophy, mental health. | p. 33–46 |
