# Supplementary material for: Navigating socio-ecological and institutional barriers to antiretroviral therapy adherence: qualitative insights among young men and women from Nairobi’s informal settlements
Source: Front Public Health. 2025 Sep 25;13:1650966. doi: 10.3389/fpubh.2025.1650966 (PMC12507891; doi:10.3389/fpubh.2025.1650966)
Supplement: Supplementary file 2 [file Supplementary_file_2.docx]

## Appendix 2: Socio-ecological model


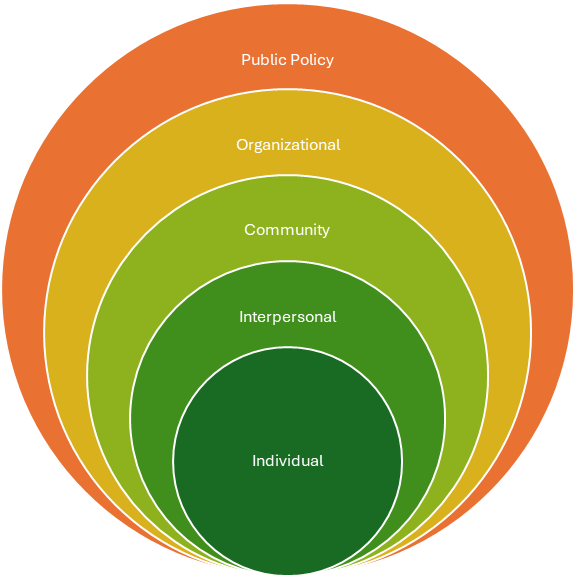


**(5) Structural barriers:** Long distances to clinics, poor infrastructure, financial barriers

**(4) Health system barriers:** Long waiting hours at clinics, lack of adolescent-friend space and privacy, negative health provider attitude

**School commitment:** Missed appointments due to tight academic schedules

**(3) Stigma & discrimination**: Fear of social rejection, community gossip, loss of friendship and social support

**Peer influence and misinformation:** Myths (erectile dysfunction, weight gain)

**(1) Negative patient attitude**: Fear of lifelong treatment, ART misinformation, forgetfulness, oversleeping

**ART knowledge;** Lack of accurate knowledge, myths (infertility, body changes

**Psychological/ behavioral factors:** Forgetfulness, drug fatigue, side effects (nausea, dizziness, hallucinations

**Physical challenges**: comorbidities (TB, Mental health

**(2) Family influences:** Lack of privacy at home leading to unintended disclosure at home

**Gender based violence:** intimate partner violence, medication sabotage by partner
