# Supplementary material for: Navigating socio-ecological and institutional barriers to antiretroviral therapy adherence: qualitative insights among young men and women from Nairobi’s informal settlements
Source: Front Public Health. 2025 Sep 25;13:1650966. doi: 10.3389/fpubh.2025.1650966 (PMC12507891; doi:10.3389/fpubh.2025.1650966)
Supplement: Supplementary file 4 [file Table_2.DOCX]

REVIEWER COMMENTS FOR FRONTIERS IN PUBLIC HEALTH JOURNAL

Manuscript: *Navigating Socio-ecological and Institutional Barriers to ART Adherence : Qualitative Insights among Young Men and Young Women in Nairobi’s Informal Settlement* .

Manuscript ID: 1650966

| **COMMENT** | **PAGE NUMBER** | **REVISED PAGE NUMBER** | **RESPONSE** |
| --- | --- | --- | --- |
| ***Reviewer 2*** |  |  |  |
| a) Additional Details on **Data Analysis (Methods Section**)  The methods section is generally well-structured but would benefit from minor clarifications:  o Provide a statement in the Methods section just before study design and setting on the use of the COREQ checklist, which is a reporting tool for qualitative studies. A sample statement would be “Study methods and findings are described per the COREQ (Consolidated Criteria for Reporting Qualitative Research) checklist. This checklist includes 32 items that must be addressed to report qualitative studies explicitly and comprehensively.” Additionally, the completed COREQ checklist should be included as part of your supplementary materials, if applicable. | 9 | 5 | This has been added on pg 5 and COREQ form as appendix 1 This study adhered to the Consolidated Criteria for Reporting Qualitative Research (COREQ) 32-item checklist (attached in Appendix 1) to ensure methodological rigor and transparency. The COREQ framework guided the reporting of key aspects including research team reflexivity, study design, participant selection, data collection, analysis, and presentation of findings. By following COREQ, we aimed to enhance credibility, reproducibility, and completeness of the qualitative methods employed, consistent with best practice standards for qualitative health research. |
| d) **Formatting and Presentation Issues**  **Grammar & Style**: Minor grammatical inconsistencies were noted. A final language proofread would enhance readability.  **Figures**  o Figure 1 needs to be redone to make the words in it legible  **References**: Double-check to ensure that all citations follow the formatting guidelines. | All through the document | All through the document  Figure 1 moved to Appendix 2 in page 47 | Grammar corrections have been done all through the document.  Figure has been reworked for better legibility of the words |
| ***Reviewer 3*** |  |  |  |
| a. **Abstract:** The study design used is not explicitly stated specify methods. The authors s should have used any of the following designs; · Phenomenological · Grounded Theory Study · Case Study | 2 | 2 | This has been edited and added to “This study employed a phenomenological design within an exploratory qualitative approach to capture and interpret the lived experiences of young adults (18–24 years) living with HIV in Kibera. Phenomenology was appropriate because it focuses on understanding participants’ subjective realities, meanings, and perceptions of ART adherence within their socio-ecological context. Through in-depth interviews, case narratives, diaries, and structured observations, the study sought to uncover how individual, interpersonal, institutional, and structural factors intersect to shape adherence behaviors from the participants’ own perspectives.” |
| b. **Chapter one**: .Page 5 par2. Citation no.12 is cited twice in the same paragraph, delete one. .Provide graphical representation of sociological model as an appendix and make in-text reference of the same. | 5 and 47 | 47 | Thank you for your observation, Page 5 par 2: One has been deleted  A graphical representation of socio ecological model has been provided as an appendix |
| c.**Chapter 2:** .Page 6 par. 1 - Study Design- Exploratory study is not a study design . Key informants- Recommended sample size was 15, but you used a less sample size i.e 10. Explain and justify why it was reduced. . Pa6 par 4- Constructionist Diary Entries-Sample size saturation point was 10 , you used 25. Why did you exceed? Provide an explanation and what implication this action has on validity of the results and conclusions drawn. .Page 8 par 2- Write IDIS in full since it is being used for the first time here. Page 10- par 3 lines 8 and 9 -Out of 132 eligible participants, a sample of 25 participants was purposively arrived at through saturation data collection......This statement is not clear. The process of selection flawed. | 4-8 | 4-8 | (Par 1) Thank you for your feedback regarding the sample size used in this study. While the recommended sample size for key informant interviews (KIIs) was 15, we conducted 10 interviews because data saturation was reached before the recommended number. That is, no new themes or insights were emerging from additional interviews, Furthermore, note, it is not unusual to reach data saturation with fewer than 10 interviews, particularly when the context is specific and participants are relatively homogeneous.   1. Guest, G., Bunce, A., & Johnson, L. (2006). How many interviews are enough? An experiment with data saturation and variability. Field Methods, 18(1), 59–82.   we purposively selected 10 key informants based on their expertise and relevance to the study context. This included a balanced mix of clinical and non-clinical healthcare providers directly involved in ART provision and adolescent/youth HIV care within Kibera. We reached thematic saturation by the 10th KII, with no new information emerging beyond this point. Additionally, the diversity of the 10 informants (clinicians, counselors, nurses, peer educators) provided a comprehensive range of perspectives on institutional and health system barriers to ART adherence, ensuring data sufficiency despite the reduced number.  (par 4) Although the recommended sample size for constructionist diary entries is approximately 10 participants, we deliberately included 25 diaries to enhance the depth, richness, and validity of our findings. This decision was informed by the need to capture the heterogeneity of experiences among young adults living in Kibera, who face diverse socio-ecological and institutional barriers to ART adherence. A larger diary sample allowed us to reflect this diversity more comprehensively, including differences by gender, school and work contexts, and treatment histories. Given the potential for incomplete diary entries, oversampling also mitigated attrition effects and ensured sufficient high-quality longitudinal data which strengthened rather than compromised validity. This approach enhanced the robustness of the findings while providing a richer, contextually grounded understanding of ART adherence among young people in informal settlements in Kibera Kenya.  (Page 10 Par 3 lines 8 and 9):  We acknowledge that the initial phrasing lacked clarity. The revised statement now explicitly outlines the selection process. “Out of the 132 eligible participants, we purposively selected a diverse sample of 25 participants to ensure representation of key sub-groups (e.g., defaulters vs. non-defaulters, males vs. females, varying treatment durations). Recruitment was guided by the principle of thematic saturation, a standard qualitative practice where sampling ceases once additional interviews no longer yield new information. This approach ensured methodological rigor by combining purposive sampling for diversity with saturation-driven recruitment, thereby strengthening the validity and trustworthiness of the findings.” |
| **c. Results-** Page 16-Out of 132 eligible participants, a sample of 25 participants was purposively arrived at through saturation data collection.How long did it take to collect data? Why is this period tied with data collection? .Page 17 - 3 participants 3(30%) were above 40 years of age..............Do not start sentence with a numeral. .Page 20- par 2. Some participants ...............state the numbers ,proportion or %. The word some is non quantifiable. . Page 20 par 3-Yes, some fear taking drugs/medicine. So, someone asks herself, “should I use these drugs for the rest of my life?” The person refuses getting linked to ART and says, “let what happens, happen.” (IDI_YMLWHIV)...... verbatim responses should be presented in rectangular textboxes with titles, numbered , italicized ,. with open and closed quotation marks , followed by code number of the respondent and and referenced in-text . Do these for all quotes. Page 30- So it depends on if they are sharing a phone with a family member whom they have not disclosed to, they might not want that but if it is their own personal phone, then they accept” (KII_HCPc......... no.? How do you differentiate respondents responses since they are not coded and numbered.? There is danger of presenting same responses from same respondent over and over again. There should be representatives in responses. You can only ensure this when the respondents are properly coded. . Your coding system is jumbled up, convoluted and confusing. Be systematic .Remove all invisible superimposed figures ,charts in some sections of the manuscript. | 16-30 | 13-30 | We appreciate your observation regarding the data collection period. The 12-month data collection period was intentional and necessary for three reasons:   1. Iterative analysis and saturation: Qualitative data collection and analysis were conducted concurrently, allowing early coding to guide subsequent interviews and confirm when saturation was reached. 2. Method triangulation**:** The extended period facilitated different qualitative methods (interviews, diaries, observations) and follow-up interactions with the same participants, providing longitudinal insights into adherence behavior. 3. Contextual constraints: Young adults in informal settlements often have irregular schedules, requiring flexible timelines for recruitment and repeat engagements to ensure adequate participation and data completeness.   Page 17: Sentence to read: “Three participants….”  We appreciate your feedback. The unique respondent numbers have been included in results section Pg 18-38 |
| **d.References** .B. Turan et al remove et.al and indicate missing year of publication., .I. Lalhruaimawii, M. V. Danturulu, S. Rai, U. K. Chandrashekar, and R. Radhakrishnan.............missing year of publication, .Remove et.all from reference list . Indicate years of publication in all ciation which do not have. |  |  | We appreciate your feedback. This has been removed Pg 65-70 |
